# Sodium Intake and Socioeconomic Status as Risk Factors for Development of Age-Related Cataracts: The Korea National Health and Nutrition Examination Survey

**DOI:** 10.1371/journal.pone.0136218

**Published:** 2015-08-19

**Authors:** Jeong Hun Bae, Doo Sup Shin, Sung Chul Lee, In Cheol Hwang

**Affiliations:** 1 Department of Ophthalmology, Kangbuk Samsung Hospital, Sungkyunkwan University School of Medicine, Seoul, Republic of Korea; 2 Department of Education and Research, Seoul National University Hospital, Seoul, Republic of Korea; 3 Department of Ophthalmology, Yonsei University College of Medicine, Seoul, Republic of Korea; 4 Department of Family Medicine, Gachon University Gil Medical Center, Incheon, Republic of Korea; The Chinese University of Hong Kong, HONG KONG

## Abstract

**Purpose:**

Cataract is a very prevalent ocular disorder, and environmental risk factors for age-related cataracts have been widely investigated. We aimed to evaluate an association of dietary sodium intake and socioeconomic factors with the development of age-related cataracts.

**Methods:**

A cross-sectional case-control study based on the 2008–2011 Korea National Health and Nutrition Examination Survey. Dietary sodium intake was estimated using urinary sodium to creatinine ratio (U[Na^+^]/Cr).

**Results:**

Among a total 12,693 participants, 2,687 (21.1%) had cataracts and 10,006 patients without cataracts served as controls. The prevalence of cataracts increased with age and quartiles of U[Na^+^]/Cr (*p* for trend < 0.001). Multivariate logistic regression analyses revealed that factors related to the development of cataracts were age ≥ 50 years (adjusted odds ratio [aOR] 15.34, 95% confidence interval [CI] 13.31‒17.69), low income (aOR 1.85, 95% CI 1.64–2.09), low educational attainment (aOR 1.76, 95% CI 1.57–1.96), and high sodium intake (U[Na^+^]/Cr > 16.4 mmol/mmol; aOR 1.29, 95% CI 1.16–1.44). In a subgroup analysis, a robust effect on cataracts across U[Na^+^]/Cr quartiles was observed in patients ≥ 50 years of age (aOR 1.11, 95% CI 1.04–1.18), though not in younger patients (aOR 1.06, 95% CI 0.96–1.17).

**Conclusions:**

Our results suggest that high sodium intake and low socioeconomic status may affect the development of cataracts, and that a low-salt diet could be helpful for the prevention of cataracts in an older population. Furthermore, efforts to close gaps in health services due to socioeconomic factors may contribute to a reduction in the prevalence of cataracts.

## Introduction

Cataracts are a major cause of blindness worldwide, affecting primarily the population 50 years of age or older [1]. Previous epidemiologic studies have established several risk factors for the development of cataracts including age, diabetes mellitus, hypertension, obesity, smoking, and low socioeconomic status (SES) [2–6]. Efforts to investigate environmental risk factors for age-related cataracts are in progress, and some studies have suggested a role of nutritional factors in the development of cataracts [3,7].

A hospital-based case-control study reported that high salt intake increased the risk of cataract extraction, with an odds ratio (OR) of 2.4 [7]. A population-based cross-sectional study also showed that higher salt intake was associated with greater risk of developing posterior subcapsular cataracts [8]. These studies suggested that sodium intake might play an important role in the pathogenesis of cataract development. Their results, however, were derived from subjective dietary questionnaires which might inaccurately measure the true amount of sodium intake [9].

It is difficult to monitor sodium intake in everyday life, and thus the measurement of 24-hour urine sodium excretion is a valuable method for estimating total sodium intake. However, 24-hour urine collection for the measurement of sodium excretion is quite cumbersome, and inaccurate collection often leads to confounded results [10]. Recent studies have shown that the urinary sodium to creatinine ratio (U[Na^+^]/Cr) obtained from a spot urine sample strongly correlates with 24-hour urine sodium excretion [11,12]. In contrast with 24-hour urine collection, the spot urine test is convenient, inexpensive, and easily performed in clinical practice. Therefore, we aimed to evaluate the association of dietary sodium intake assessed using the U[Na^+^]/Cr and socioeconomic factors with the development of cataracts in the Korean population.

## Materials and Methods

### Data Source and Study Participants

This study was based on data from the Korea National Health and Nutrition Examination Survey (KNHANES) 2008–2011, which is a nationwide population-based survey that is regularly performed by the Korean Ministry of Health and Welfare. This is the second and third years of the fourth (IV-2, 3) combined with the first and second years of the fifth (V-1, 2) study that uses a stratified, multistage probability sampling design for the selection of household units. Individuals were randomly selected for inclusion in the KNHANES according to sampling units based on age data drawn from household registries, economic status, sex, and geographical area [13]. In 2008–2011, a total of 46,777 individuals greater than 1 year of age were included in the survey, and of these, 37,753 individuals participated in the health examination survey (overall response rate, 80.7%).

The survey had four components: the Health Interview Survey, the Health Behavior Survey, the Health Examination Survey, and the Nutrition Survey. All interviews and examinations were conducted in specially designed and equipped mobile centers that traveled throughout the country. Surveys were conducted using self-administered questionnaires, and interviewers were available to assist those participants who had difficulty with self-administration. Interviewers were not given any information about the specific participants in advance, and all participants provided written informed consent to participate in the study. The KNHANES studies were conducted according to the guidelines put forth in the Declaration of Helsinki. This study was approved by the Institutional Review Board of Kangbuk Samsung Hospital (IRB No. KBSMC 2014-12-002). Detailed information on the survey design has been described elsewhere [12,14].

We identified 30,401 participants who underwent ophthalmic examination. Of these, we excluded 17,708 participants due to the following: (i) age < 19 years, (ii) chronic diseases (i.e., stroke, myocardial infarction, liver disease, and malignancy), (iii) a history of diabetes mellitus or prior ocular surgery except cataract extraction [15], (iv) taking antihypertensive medications [12], and (v) renal disease or impaired renal function (serum creatinine > 1.2 mg/dl) [12]. A history of diseases was defined by the presence of a physician’s diagnosis. Subjects who had no available data on urinary sodium or creatinine levels or the presence of cataracts were also excluded.

### Data Collection

Information on demographic characteristics (age, sex, household income, educational attainment, and marital status), health behaviors (physical activity, smoking history, and alcohol consumption), and history of dyslipidemia diagnosed by physician was collected during the health interview. Health behaviors were assessed using questions about these habits during a one-month period before the interview. After the interview, height and body weight were measured with the participants wearing light clothing and no shoes. Body mass index (BMI) was calculated as weight in kilograms divided by the square of height in meters.

Obesity was defined as a BMI ≥ 25 kg/m^2^ according to the Korean Society for the Study of Obesity. Other variables were categorized as follows: economic status was classified into quartiles according to mean monthly household income, which was calculated by dividing household income by the square root of the number of persons in the household [16], education attainment was categorized as “middle school or lower” or “high school or beyond”, marital status was classified as “married” or “unmarried” which included “single” and “divorced/separated/widowed”, physical activity was classified as “regular physical activity” when participants engaged in moderate-intensity activity more than 5 times per week or vigorous activity more than 3 times per week or “other” based on the International Physical Activity Questionnaire short-form scoring protocol [17], smoking status was classified as “non-smoker” or “past or current smoker”, alcohol consumption was categorized as “problem drinker”, which was defined as more than 7 drinks (men) or 5 drinks (women) in one sitting and more than 2 days per week, or “other”.

As a part of the standardized ophthalmic examination, lens status was evaluated with slit-lamp biomicroscopy (Haag-Streit model BQ-900, Haag-Streit AG, Koeniz, Switzerland) by certified, experienced ophthalmologists using standard photographs from the Lens Opacities Classification System III (LOCS III) [18]. Lens opacities were classified as cortical (LOCS III score ≥ 2 for cortical cataracts), nuclear (LOCS III score ≥ 4 for nuclear opalescence/color), posterior subcapsular (LOCS III score ≥ 2 for posterior subcapsular cataracts), and mixed cataracts (more than one type per eye) [19]. The presence of cataracts was defined as any one or more types of lens opacity or evidence of cataract extraction in at least one eye, while absence of cataract was defined in participants with no evidence of lens opacity or prior cataract surgery. The quality of the ophthalmic survey and cataract evaluation was verified by the Epidemiologic Survey Committee of the Korean Ophthalmologic Society [20].

Spot urine samples were collected in the morning after fasting for at least 8 hours. Analysis of spot urine levels of sodium and creatinine was performed using a Hitachi Automatic Analyzer 7600 (Hitachi Ltd., Tokyo, Japan), and U[Na^+^]/Cr was calculated (mmol/mmol).

### Statistical Analyses

All statistical analyses were performed using STATA SE 9.2 (STATA Corp., TX, USA), which incorporates sample weights and adjusts the analyses for the complex sample design of the survey. Survey sample weights were used to produce estimates that are representative of the non-institutionalized civilian Korean population.

We used independent t-tests or χ^2^ tests to estimate differences in the characteristics of participants according to the presence of cataracts. We used the logistic regression model to identify factors related to the development of cataracts. Each independent variable that was statistically significant on univariate analysis was evaluated in the final multivariate logistic model. Furthermore, to assess its association with the development of cataracts, U[Na^+^]/Cr was categorized by quartiles as follows: Q1 (≤ 6.8), Q2 (6.8–10.7), Q3 (10.7–16.4), and Q4 (> 16.4). The ORs and 95% confidence intervals (CIs) for cataract development were calculated for each quartile before and after adjusting for potential confounding variables. These associations were also assessed in multiple logistic regression analyses using log_2_-transformed U[Na^+^]/Cr as a continuous variable. All statistical tests were two-sided, and statistical significance was determined at *p* < 0.05.

## Results

### Characteristics of Participants

**Fig 1:**
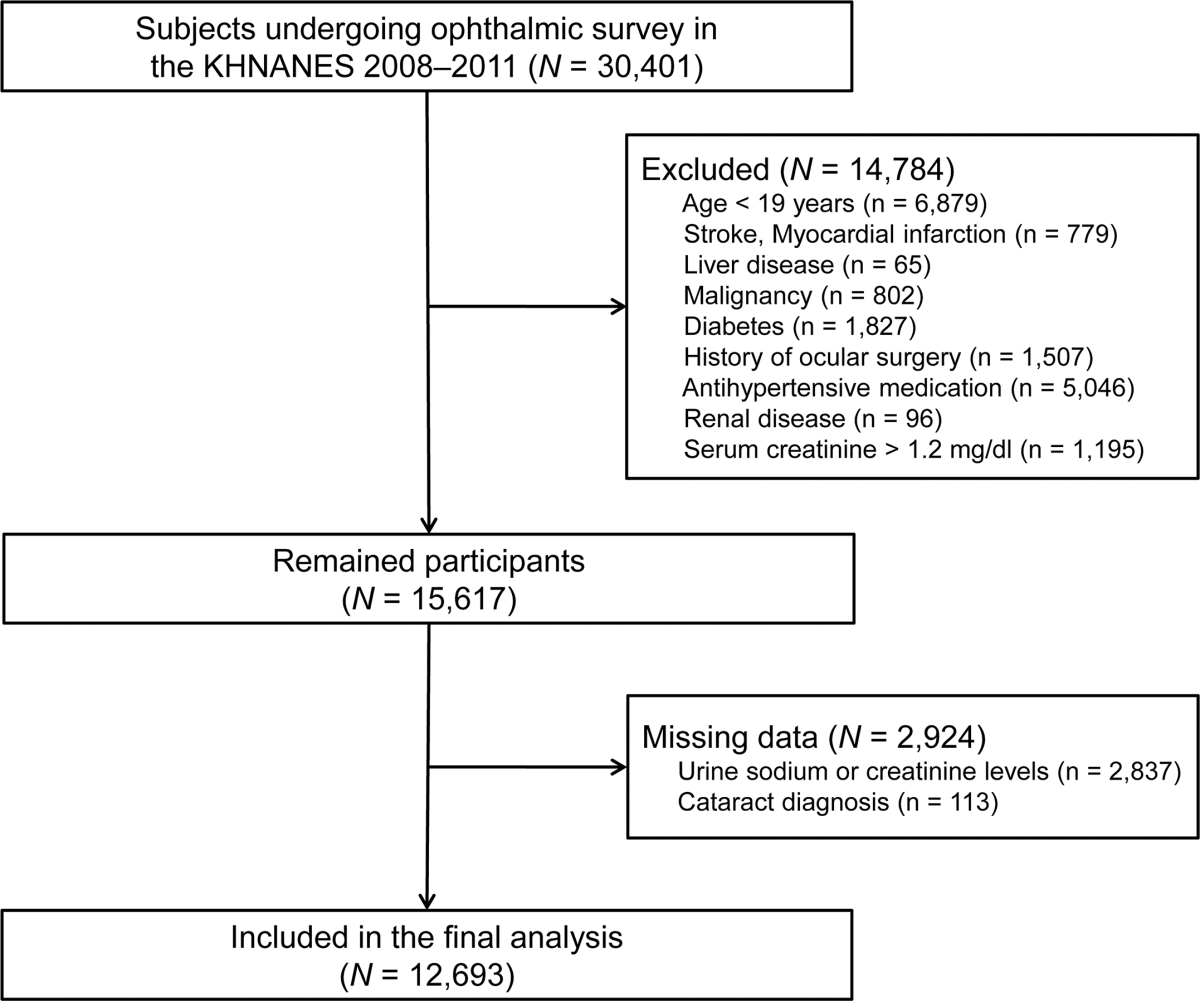
Flow diagram of study participants.

Of the 30,401 eligible participants, 12,693 were included in the final analysis (Fig 1). Table 1 shows the characteristics of participants stratified by the presence or absence of cataracts. Participants in the cataract group were older than those in the non-cataract group (62.3 ± 10.9 vs. 40.0 ± 11.5 years, *p* < 0.001). In the cataract group, the proportions of participants with low economic or educational status, those that were married, past smokers, and those with dyslipidemia were much higher than those observed in the non-cataract group. The mean value of U[Na^+^]/Cr was significantly higher in the cataract group than in the non-cataract group (16.2 ± 9.5 vs. 11.7 ± 7.6 mmol/mmol, *p* < 0.001). We further analyzed the data according to subtypes of cataracts: cortical (n = 579), nuclear (n = 1,563), and posterior subcapsular cataracts (n = 110). However, no significant differences in the median value of U[Na^+^]/Cr were found among subtypes (*p* = 0.836).

**Table 1:** Characteristics of participants with and without cataracts.

	Non-cataract	Cataract	
	(*n* = 10,006)	(*n* = 2,687)	*p-*value
Age, years (mean ± SD)	40.0 ± 11.5	62.3 ± 10.9	<0.001
Sex, % of female	54.6	53.9	0.479
Household income, %			<0.001
Very low	9.6	32.3	
Low	24.9	26.7	
Moderate	33.0	21.8	
High	32.6	19.3	
Education, %			<0.001
≤ Middle school	17.6	65.1	
≥ High school	82.4	34.9	
Marital status, % married	72.1	77.2	<0.001
Smoking status, %			<0.001
Never	56.8	56.8	
Past	9.5	13.1	
Current	33.8	30.1	
Problem drinker, %	5.2	5.7	0.293
Regular physical activity, %	23.8	23.8	0.977
Obesity, %	28.4	26.9	0.122
Dyslipidemia, %	3.8	8.1	<0.001
Serum sodium, mmol (mean ± SD)	129.6 ± 54.5	139.5 ± 49.8	<0.001
U[Na^+^]/Cr, mmol/mmol (mean ± SD)	11.7 ± 7.6	16.2 ± 9.5	<0.001

SD, standard deviation; U[Na^+^]/Cr, urinary sodium to creatinine ratio.

*P*-values were calculated using t-tests or χ^2^ tests.

### Factors Related to Development of Cataracts

Table 2 lists the factors related to the development of cataracts. On univariate analysis, participants with age ≥ 50 years (OR 24.48, *p* < 0.001), low household income (OR 2.73, *p* < 0.001), low educational attainment (OR 8.74, *p* < 0.001), unmarried status (OR 0.76, *p* < 0.001), dyslipidemia (OR 2.25, *p* < 0.001), and high sodium intake (OR 2.71, *p* < 0.001) were more likely to develop cataracts. On multivariate analysis, the significance of high sodium intake remained robust (adjusted OR [aOR] 1.29, *p* < 0.001), along with older age (aOR 15.34, *p* < 0.001), low household income (aOR 1.76, *p* < 0.001), and low educational attainment (aOR 1.85, *p* < 0.001).

**Table 2:** Odds ratios and 95% confidence intervals for associations between various demographic and clinical factors and the development of cataracts.

	Univariate (*n* = 12,693)			Multivariate (*n* = 12,438)		
	OR	95% CI	*p-*value	aOR	95% CI	*p-*value
Age (≥ 50 years old)	24.48	21.63–27.71	<0.001	15.34	13.31–17.69	<0.001
Female	0.97	0.89–1.06	0.479			
Low household income	2.73	2.50–2.98	<0.001	1.76	1.57–1.96	<0.001
Low education level	8.74	7.95–9.61	<0.001	1.85	1.64–2.09	<0.001
Unmarried	0.76	0.69–0.84	<0.001	1.03	0.91–1.18	0.626
Obese	0.93	0.84–1.02	0.122			
Smoker	1.00	0.92–1.09	0.987			
Problem drinker	1.11	0.92–1.33	0.293			
Non-exerciser	1.00	0.91–1.11	0.977			
Dyslipidemia	2.25	1.89–2.67	<0.001	1.15	0.94–1.41	0.182
High sodium intake^*^	2.71	2.48–2.97	<0.001	1.29	1.16–1.44	<0.001

CI, confidence interval; OR, odds ratio.

*P* values were obtained using a logistic regression model.

*>75% (16.4 mmol/mmol) of urinary sodium to creatinine ratio in the current sample.

### Cataracts and U[Na^+^]/Cr

**Fig 2:**
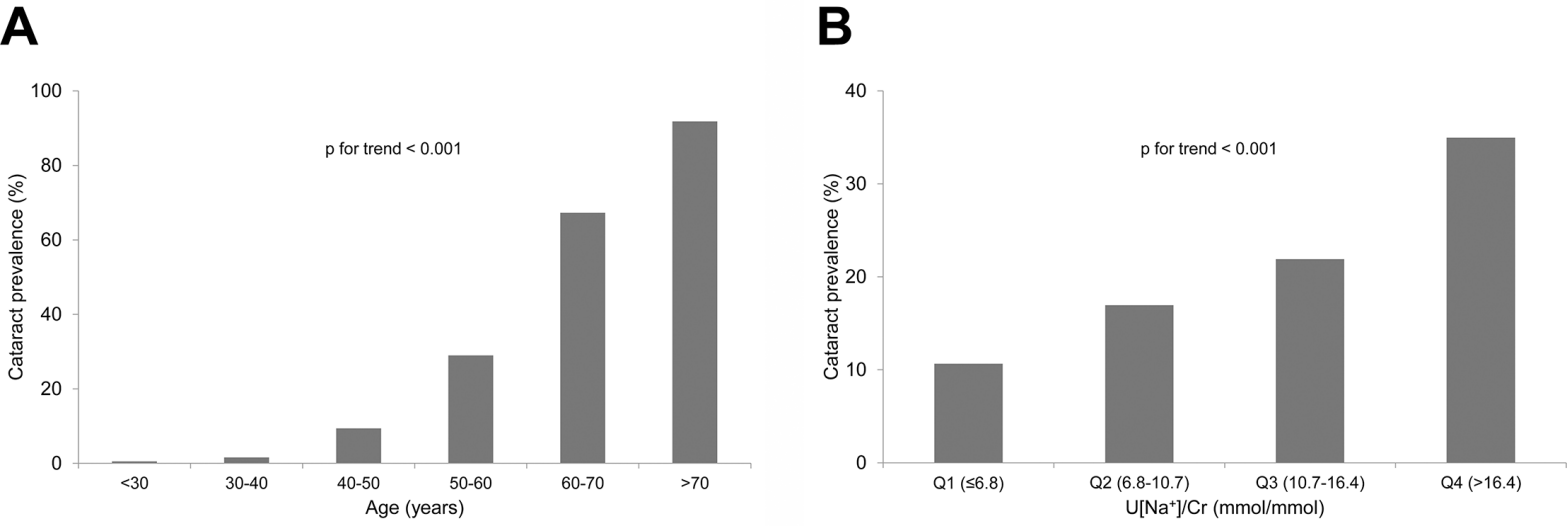
The prevalence of cataracts across age groups (A) and quartiles of urinary sodium to creatinine ratios (U[Na^+^]/Cr) (B).

The prevalence of cataracts was significantly increased according to age and quartile of U[Na^+^]/Cr (both, *p* for trend < 0.001) (Fig 2). Table 3 shows the ORs and 95% CIs for developing cataracts according to quartiles of U[Na^+^]/Cr in different age groups. In contrast to the younger group, the group ≥ 50 years of age exhibited a significant association between a high U[Na^+^]/Cr and the risk of developing cataracts, and the aOR for doubling U[Na^+^]/Cr was 1.13 (95% CI 1.05–1.22, *p* < 0.01). On logistic regression analysis using quartiles of U[Na^+^]/Cr, the significance of trends across quartiles (aOR 1.11, 95% CI 1.04–1.18; *p* < 0.01) and in quartile 4 (aOR 1.12, 95% CI 1.04–1.19; *p* < 0.01) was found after adjusting for potential confounders in patients ≥ 50 years of age.

**Table 3:** Odds ratios and 95% confidence intervals for developing cataracts according to quartiles of urinary sodium to creatinine ratios (U[Na^+^]/Cr) in different age groups.

	< 50 years old		≥ 50 years old	
	Unadjusted (*n* = 8,111)	Adjusted^†^ (*n* = 7,972)	Unadjusted (*n* = 4,582)	Adjusted^†^ (*n* = 4,466)
Per doubling of U[Na^+^]/Cr	1.14 (1.01–1.29)^*^	1.09 (0.97–1.24)	1.24 (1.16–1.32)^***^	1.13 (1.05–1.22)^**^
U[Na^+^]/Cr quartiles, mmol/mmol				
Quartile 1 (< 6.8)	1	1	1	1
Quartile 2 (6.8–10.7)	1.24 (0.93–1.66)	1.21 (0.90–1.61)	1.19 (0.97–1.48)	1.17 (0.94–1.46)
Quartile 3 (10.7–16.4)	1.04 (0.89–1.21)	1.02 (0.87–1.19)	1.12 (1.01–1.23)^*^	1.07 (0.97–1.19)
Quartile 4 (> 16.4)	1.13 (1.01–1.26)^*^	1.08 (0.97–1.21)	1.19 (1.12–1.27)^***^	1.12 (1.04–1.19)^**^
*p* for trend	1.09 (0.99–1.21)	1.06 (0.96–1.17)	1.19 (1.12–2.26)^***^	1.11 (1.04–1.18)^**^

*p<0.05

**p<0.01

***p<0.001; *p* values were obtained using a logistic regression model.

^†^Adjusted for economic status, educational level, marital status, and dyslipidemia.

## Discussion

In this nationwide population-based survey, subjects ≥ 50 years of age with a high U[Na^+^]/Cr had a significantly higher prevalence of cataracts compared to those with low U[Na^+^]/Cr. We initially excluded subjects who had potential risk factors for cataract development. Thus, this study demonstrates that high sodium intake may increase the risk of cataract development in apparently healthy subjects, which is in line with previously reported results. Our findings, however, should be interpreted cautiously since the pathophysiology of this association in an older population is unclear. It may be the case that elderly people may be more vulnerable to salty diets compared with younger people in the development of cataracts.

An Italian hospital-based study of 207 patients who underwent cataract surgery reported significantly higher sodium intake in the study group compared with the control group [7]. In addition, the Blue Mountain Eye Study, which was a population-based cross-sectional study (n = 2,873), similarly showed that a high-salt diet might increase the risk of developing posterior subcapsular cataracts [8]. Individual sodium intake in those studies was based on assessment of self-reported dietary questionnaires.

Our study had several strengths over previous studies. First, this was a large population-based study, and all participants came from an ethnically homogenous population which may have reduced racial variability. Second, dietary sodium intake was evaluated using spot urine analysis for U[Na^+^]/Cr, which correlates well with 24-hour urine sodium excretion. This method may provide a more accurate and objective estimation of individual sodium intake than subjective dietary questionnaires [11,12]. Finally, clinical ophthalmic data were collected independently from demographic data, health behaviors, medical history, and laboratory studies. This independent data collection might reduce the potential for bias.

The mechanism of cataractogenesis due to high sodium intake is unclear, though an animal study using Dahl salt-sensitive rats suggested that a marked Na^+^/K^+^ electrolyte imbalance in the aqueous humor and the lens was significantly associated with the formation of cataracts [21]. An imbalance in serum sodium levels caused by a chronic high-salt diet led to a rise in the aqueous humor and lenticular sodium concentrations, which might exceed the capacity of the Na^+^/K^+^ channels in the lens and result in the expansion of extracellular fluid volume and development of lens opacities [22]. This volume-related mechanism is quite similar to that responsible for systemic hypertension, which is a known risk factor for developing cataracts. From this perspective, however, the risk for cataract development is not directly increased by systemic hypertension, but rather both hypertension and cataracts may be the consequence of the expansion in extracellular fluid volume induced by high sodium intake. This is supported by evidence that the restriction of dietary sodium intake prevented the development of hypertension and cataracts with normalization of the lenticular and aqueous humor sodium concentrations in Dahl salt-sensitive rats [23].

In our study, socioeconomic factors such as low household income and educational attainment were strongly associated with the prevalence of cataracts. Taken together with previous findings, it could be speculated that people with low SES may have difficulties accessing medical services and obtaining information on health risk prevention [24,25]. Therefore, the rates of cataract surgeries are presumed to be lower in people with low SES, which may contribute to higher cataract prevalence [26].

Given that socioeconomic factors are closely related to dietary patterns, SES should be considered as a potential confounder when evaluating the association between dietary intake and the development of cataracts [3,27]. People with low SES tend to have poorer dietary habits such as high fat and sugar diets, and several studies have reported an association of hypertriglyceridemia and hyperglycemia with the development of cataracts [28–31]. We also found that higher serum triglyceride and total cholesterol levels and lower high-density lipoprotein levels were associated with the presence of cataracts in the study population (individual data not shown), although this association did not persist after adjustment. As people with diabetes were excluded from the study, the effects of hyperglycemia might be attenuated in our results. A recent study in a Japanese population reported that low SES was significantly associated with high salt intake, which has been suggested as a risk factor for cataract formation in our study as well as in earlier epidemiologic studies [32]. Thus, both high sodium intake and low SES could be confounding factors to each other in analyzing their association with cataracts. Our multivariate analysis results, however, showed that both high sodium intake and low SES are independent risk factors for the development of cataracts.

Previous epidemiologic studies have suggested that smoking is associated with a high prevalence of cataracts [26,33,34]. In this study, however, we could not identify an association between smoking history (including both current and past smokers) and cataract prevalence. The relationship might be affected by the duration or amount of smoking, but these quantitative data were not available for analysis. This discrepancy may also be derived from the ethnic differences, random variation in study populations, or different study designs (i.e., a greater number of past smokers in the cataract group may indicate the reverse causality), though further investigation is needed.

We observed no significant differences in the prevalence of cataract according to sex. In both the United States and Korea the prevalence of cataract is reported to be higher in females than in males, while no significant differences are observed in Myanmar or China [20,35–37]. This disparity among studies might be due to differences in study samples or designs. To assess the relationship between sodium intake and cataract, we excluded subjects with factors that could affect sodium intake or excretion or that would increase the risk of cataracts. As a result, sex differences regarding the prevalence of cataract might be diluted throughout the exclusion process.

This study has several limitations. First, its cross-sectional design does not permit us to infer causal relationships between cataract development and risk factors. Further prospective research is warranted to determine causality. Second, ocular examinations were performed by multiple examiners because the KNHANES is a large population-based study, and therefore the possibility of misclassification cannot be excluded. In addition, as data regarding some of the diseases addressed in this study were collected using an interviewer-administered questionnaire, there may be reporting errors resulting from recall bias. Third, although U[Na^+^]/Cr seems to be correlated with the measurement of 24-hour sodium excretion for assessing dietary sodium intake, there are limitations of using U[Na^+^]/Cr such as temporal variation of sodium excretion and individual differences in 24-hour creatinine excretion [11,38]. Fourth, several risk factors for cataracts, such as ultraviolet light exposure, dietary antioxidant intake, and the number of children born (in females) were not investigated in this study. Despite these limitations, this study’s findings are highly informative. We identified a significant association of sodium intake and SES with the development of cataracts in patients ≥ 50 years of age. Our results provide further evidence that a low-salt diet may be helpful for the prevention of cataracts. In addition, efforts to close gaps in access to health services according to the SES may contribute to a reduction in the prevalence of cataracts. Well-designed prospective studies and collaborative research to confirm these relationships are warranted.
